# Social media use, economic recession and income inequality in relation to trends in youth suicide in high-income countries: a time trends analysis

**DOI:** 10.1016/j.jad.2020.05.057

**Published:** 2020-10-01

**Authors:** Prianka Padmanathan, Helen Bould, Lizzy Winstone, Paul Moran, David Gunnell

**Affiliations:** a Population Health Sciences, Bristol Medical School, University of Bristol, Bristol, UK; b National Institute of Health Research Biomedical Research Centre at the University Hospitals, Bristol NHS Foundation Trust and the University of Bristol, Bristol, UK

**Keywords:** Suicide, Youth, Social media, Recession, Inequality

## Abstract

**Background:**

Suicide rates have risen in young people in several high-income countries over the last decade. Reasons for the increases are unclear.

**Methods:**

We analysed trends in suicide rates in 15-24 year olds over the period 2000-2017 in high-income countries with populations >20 million using Joinpoint analysis. We investigated differences in the following population-level exposures between countries that are and are not experiencing suicide rates rises: 1) 2008 economic recession as indexed by changes in GDP; 2) Gini income inequality; 3) daily social media use.

**Results:**

Four of the 11 countries studied are experiencing youth suicide rate rises: Australia, Canada, the UK, the USA. The year the increase began ranged from 2003 (95% confidence interval: 2002, 2007) in the UK to 2009 (95% CI: 2007, 2012) in Australia. There was little evidence of an association between social media use and youth suicide trends, and inconsistent evidence regarding the impact of the 2008 economic recession. Suicide rate rises were seen in countries with higher GDP per capita (Wilcoxon rank sum (WRS) z=-2.27; p=0.02) and income inequality (WRS z=-2.45; p=0.01) in 2008.

**Limitations:**

Suicide data were only available until 2016/2017. Social media and income inequality data were not available for all study years. The effect of other important factors were not investigated due to a lack of comparable data.

**Conclusions:**

Our analyses indicate that the most populous high-income countries experiencing a rise in youth suicide rates are predominantly English-speaking, with higher levels of income inequality and GDP. These findings provide preliminary evidence regarding possible contributory factors to guide further research.

## Highlights

• We investigated 2000-2017 youth suicide trends in populous high-income countries.
• 4 of the 11 study countries are experiencing a rise in youth suicide rates.
• There was little evidence of an association between social media use.
• Evidence of an association with the 2008 economic recession was inconsistent.
• The rises were associated with some measures of income inequality and GDP.

## 1 Introduction

Suicide rates in young people have markedly increased in several high-income countries over the last decade (Bould et al., 2019; Ruch et al., 2019; Stefanac et al., 2019). In England and Wales, for example, rates almost doubled in 15-19 year olds between 2010 and 2017, with some evidence that the rise was more marked in females than males (Bould et al., 2019).

These increases appear to have been accompanied by other signals of deteriorating mental health among young people (McManus et al., 2014; McManus et al., 2019; Twenge et al., 2019). For example, the prevalence of common mental disorders and self-harm in the United Kingdom (UK) rose between 2000-2014, particularly among 16-24 year old females (McManus et al., 2014; McManus et al., 2019).

In addition to enhancing investment in mental health services for young people, there is a pressing need for appropriate population-based prevention strategies (Gunnell et al., 2018). To achieve this, we need a better understanding of the drivers of suicide trends among young people. Yet, whilst there has been much speculation about possible contributors to recent trends, empirical evidence is lacking. Possible explanations for the rise include widening social inequality, the 2008 global recession and associated austerity measures, as well as a wide range of disparate sources of stress now facing young people. These stresses include greater academic pressures, increasing social media use, rising rates of family instability, growing concerns about the environment, and an increase in the rates of gambling and drug dependence. For factors where comparable data exists, a cross-national analysis of temporal changes in these factors and their relation to changes in youth suicide rates, could assist in the identification of tractable risk factors underlying youth suicide.

We investigated trends in suicide rates among 15-24 year olds in the most populous high-income countries since 2000. High-income countries were selected because recent studies have highlighted that some of the most striking rises in suicide rates have occurred in these settings (Bould et al., 2019; Ruch et al., 2019; Stefanac et al., 2019), and some of the key drivers of recent trends in suicide rates differ between low- and high-income countries, for example the prevalence of underlying mental health problems in the population (Knipe et al., 2019). As suicide rates in 15-24 year olds tend to be lower than in other age groups, leading to instability in rate trends in smaller countries, we focused on countries with populations of greater than 20 million. We investigated concurrent trends in three leading contenders as explanations for suicide rate rises, which were selected on a priori grounds based on previously published associations with suicidal behaviour: the 2008 global recession, increases in income inequality, and growth in social media use.

We hypothesised that: a) if rises in suicide rate are influenced by the 2008 economic recession, they will have occurred exclusively in countries experiencing a recession, at the time of the recession, as indexed by changes in gross domestic product (GDP); b) if income inequality influences suicide rates, rises in suicide rates will have occurred in countries with greater absolute, or increasing, income inequality; c) if rises in suicide rates are associated with growth in social media use, all countries will have experienced a rise in the incidence of suicide in 15-24 year olds, and increases in suicide will be greatest in countries that have experienced the greatest increases in use.

## 2 Methods

### 2.1 Outcome data

We used World Bank data (2019 revision) to identify high-income countries with total populations of 20 million people or more (World Bank, 2019) (Web Appendix 1). This cut point was selected pragmatically to balance the need for sufficient power to detect trends, whilst ensuring graphical presentation of the data remained manageable. The countries with total populations of at least 20 million were: Australia, Canada, France, Germany, Italy, Japan, Poland, Saudi Arabia, Republic of Korea, Spain, UK, USA. These countries are home to approximately 78% of all people living in high-income countries worldwide (Web Appendix 1). We extracted annual population data for 15-24 year olds from the year 2000 until 2017, the most recent year for which suicide data were available.

The World Health Organisation's (WHO) mortality statistics raw data files (Dec 2019 update) were used to extract country-specific data on the number of suicides in 15-24 year olds since 2000, until the most recent year for which data were available (between 2016-2017, Web Appendix 2), for the countries listed above (World Health Organisation, 2019). Data were unavailable for 2000-2002 and 2000 for Italy and the UK respectively, so data from the WHO mortality database (May 2018 update) were used for these time points instead (World Health Organisation, 2018). Age-specific suicide data for Saudi Arabia were not recorded on the WHO database for most of the study years and so it was excluded from the analysis.

### 2.2 Exposure data

We used World Bank data on GDP per capita in international dollars, based on purchasing power parity, as our indicator of recession and recovery (World Bank, 2017). A fall in GDP in two consecutive quarters is a widely used definition of economic recession. For simplicity, and to identify gross changes in GDP, we used annual GDP data and identified recession as at least one year of falling GDP. Data were extracted for each of the eleven countries studied from 2000 to 2017.

We also used World Bank data on the Gini index, a widely used measure of income inequality, where zero represents perfect income equality and 100 represents perfect inequality (World Bank, 2017). For most countries, there was no complete time series data available for the Gini index (Web Appendix 2). We therefore extracted estimates for the three years (2004, 2008 and 2014) covering periods close to the beginning, midpoint and end of the time period examined, for which data were available for most countries. Where data for these years were missing, we used data for the nearest available year. For Japan, Gini index data were only available for a single year (2008).

There are limited international data on secular trends in social media use spanning our study period. We used data from GlobalWebIndex https://www.globalwebindex.com/. The company has collected survey data every three months on social media usage for all study countries since quarter 4 of 2012. Annual sample sizes of 16-64 year olds from each country range from 1,500-4,000 in 2012 up to 5,000-40,000 in 2019. Participants, whose email addresses are verified, are recruited via a range of online methods such as advertising and messaging. To ensure that the research is representative of each country's online population, quotas are set on age, gender and education. Participants are asked, in their local language, to categorise their average daily social media use into one of nine categories ranging from no use up to more than 10 hours per day.

There is no consensus on appropriate levels of daily social media use. A recent analysis of data from the UK Millennium cohort at age 14 (in 2014-16) reported that 43% of girls and 22% of boys used social media for more than three hours daily (Kelly et al., 2018). The authors reported that depression symptom scores for those with three to five hours daily use were 26% higher in girls and 21% higher in boys compared with those using social media for less than three hours daily; for ≥5 hours use the equivalent figures were 50% in girls vs 35% in boys. As data on ≥5 hours were unavailable, we extracted annual data on the proportion of 16-24 year olds from each study country using social media for an average ≥3 hours per day. Annual data on ≥1 and ≥6 hours social media use were also extracted for sensitivity analyses.

### 2.3 Data analysis

Annual country-specific suicide rates per 100,000 and standard errors were calculated based on the number of suicide deaths of people aged 15-24 years old and the total population aged 15-24 years old in each country for each year between 2000 and the most recent year for which data were available (between 2016-2017). Trends, and the timing of changes in trends (“joinpoints”), were then investigated using the US National Cancer Institute's Joinpoint trend analysis software (version 4.7.0.0) (National Cancer Institute, 2019).

GDP and the percentage of people using social media for ≥1, ≥3 hours and ≥6 hours daily were graphed for each country, and trends were related to visual inspection of trends in suicide rates. We did not undertake a formal time series analysis due to the short time period studied (17 years) and limited data for social media use and GINI index for much of the study period.

As social media use survey data were only available for the last quarter of 2012 and two quarters in 2019 and suicide data only available until 2016/17, we focused on pooled quarterly data for 2013-2017 in our analysis.

Annual social media use data were only available from 2013 onwards, yet upturns in suicide rates in study countries occurred before 2013. To investigate social media use prior to the upturns in suicide rates in these countries, we considered the prevalence of social media use in 2013 as a proxy for the extent of growth in use over the preceding decade, noting that social media use is likely to have been almost 0% before 2003.

For each hypothesised exposure we compared countries where suicide rates are and are not rising, using Wilcoxon rank sum tests (GDP, income inequality and social media use) or Fisher's exact test (economic recession). All analyses were conducted using Stata version 15MP (Statacorp, 2017).

## 3 Results

**Fig. 1:**
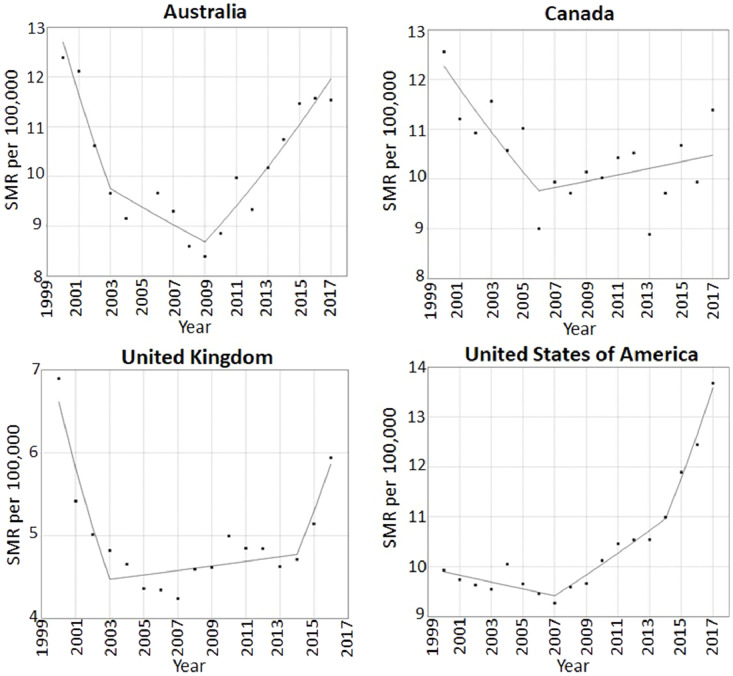

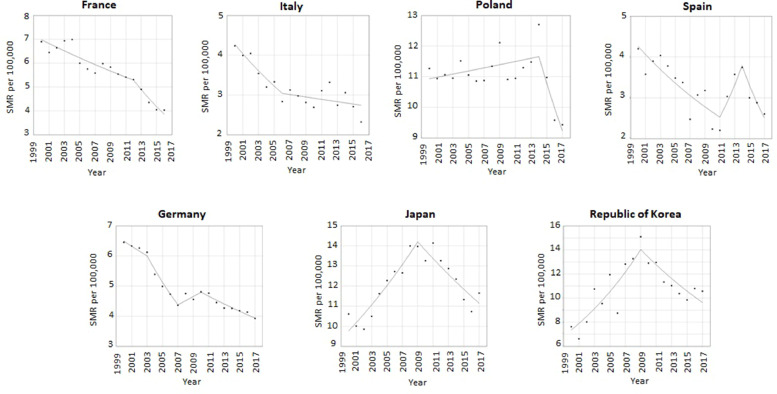
a. Suicide rates per 100,000 in 15-24 year olds in high-income countries experiencing a rise in suicide rates. b: Suicide rates per 100,000 in 15-24 year olds in high-income countries not experiencing a rise in suicide rates.

Suicide rates are rising in Australia, Canada, the UK and USA (Table1/ Fig. 1). The year the increase began ranged from 2003 (95% confidence interval: 2002, 2007) in the UK to 2009 (95% CI: 2007, 2012) in Australia. The greatest current increases are in UK (10.8% per year, 95% CI: -3.8%, 27.8%) and USA (7.4% per year, 95% CI: 4.6%, 10.3%) and the smallest is in Canada (0.6% per year, 95% CI: -0.5, 1.9). Sex-stratified analysis indicated that suicide rates are rising in both males and females in Australia, the UK and the USA, although in the USA rates in females have risen continuously throughout the study period without a preceding decline. In contrast, statistical evidence for a rise was only seen amongst females in Canada (Web Appendix 3 & 4).

**Table 1:** Summary of annual percent change (APC) in suicide rates (2000-2017) and joinpoints (years when change in trend were detected), with 95% confidence intervals (CI), for modelled trends in suicide rates in 15-24 year olds in high-income countries

Country	Segment 1 APC, suicide rate (95% CI)	Joinpoint between segments 1 & 2, Year (95% CI)	Segment 2 APC, suicide rate (95% CI)	Joinpoint between segments 2 & 3, Year (95% CI)	Segment 3 APC, suicide rate (95% CI)	Joinpoint between segments 3 & 4, Year (95% CI)	Segment 4 APC, suicide rate (95% CI)
*Countries where suicide rates are rising*							
**Australia**	-8.4 (-14.5, -1.9)	2003 (2002, 2006)	-1.9 (-5.3,1.6)	2009 (2007, 2012)	4.1 (2.6, 5.6)		
**Canada**	-3.7 (-6.5, -0.9)	2006 (2003, 2015)	0.6 (-0.5, 1.9)				
**UK**	-12.2 (-18.3, -5.7)	2003 (2002, 2007)	0.6 (-0.6, 1.8)	2014 (2005, 2014)	10.8 (-3.8, 27.8)		
**USA**	-0.7 (-1.5, 0.1)	2007 (2005, 2010)	2.2 (1.2, 3.2)	2014 (2012, 2015)	7.4 (4.6, 10.3)		
*Countries where suicide rates are not rising*							
**France**	-2.3 (-3.2, -1.4)	2012 (2007, 2014)	-7.5 (-12.7, -2.1)				
**Germany**	-2.6 (-5.0, -0.1)	2003 (2002, 2005)	-7.6 (-10.1, -5.0)	2007 (2005, 2008)	3.1 (-2.7, 9.3)	2010 (2008, 2015)	-2.8 (-3.6, -2.0)
**Italy**	-5.5 (-8.9, -1.9)	2006 (2002, 2010)	-1.0 (-3.0, 1.0)				
**Japan**	4.3 (2.9, 5.6)	2009 (2007, 2012)	-3.0 (-4.6, -1.3)				
**Poland**	0.5 (0.0, 0.9)	2014 (2013, 2015)	-7.5 (-12.8, -1.8)				
**Republic of Korea**	7.4 (4.2, 10.8)	2009 (2006, 2011)	-4.6 (-7.9, -1.2)				
**Spain**	-4.6 (-6.7, -2.5)	2011 (2008, 2012)	14.4 (-19.4, 62.5)	2014 (2012, 2015)	-12.8 (-28.1, 5.7)

[Fig. 1]

In 2009 both Japan and South Korea experienced downturns in suicide rates around the time of the upturns in other countries. France and Italy have experienced consistent declines in suicide rates over the study period, although the rate of decline has increased in France and decreased in Italy. Germany and Spain both experienced temporary increases in suicide rates in 2007-10 and 2011-14 respectively, followed by a return to a downward trend.

### 3.1 Suicide trends in relation to the 2008 global recession

All four countries where youth suicide rates are rising had economic recessions in 2008-9 (Table 2, Web Appendix 5). The drop in GDP per capita in Australia was, however, negligible (0.1% compared with 4.1-6.0% in Canada, the UK and the USA). The upturn in youth suicide in three of the four countries appear to have predated the economic recessions, although the 95% confidence intervals for USA and Canada are consistent with it beginning around the time of the recession. Furthermore, both the UK and the USA experienced an acceleration in the rise in suicide rates after the economic recession.

**Table 2:** **Timing of upturns in suicide rates with 95% confidence intervals (CI) in relation to evidence of recession** (hypothesis: if rises in suicide rate were influenced by the 2008 global economic recession, they will have occurred exclusively in countries experiencing a recession as indexed by a fall in GDP).

Country	Year of upturn in suicide rates (95% CI)	Year and duration of economic recession
*Countries where suicide rates are rising*		
**Australia**	2009 (2007, 2012)	Minor drop in GDP per capita in 2009
**Canada**	2006 (2003, 2015)	2008-2009
**UK**	2003 (2002, 2007) Further increase in upward suicide rate trend: 2014 (2005, 2014)	2008-2009
**USA**	2007 (2005, 2010) Further increase in upward suicide rate trend: 2014 (2012, 2015)	2008-2009
*Countries where suicide rates are not rising*		
**France**		2008-2009
**Germany**	Temporary upturn: 2007 (2005, 2008)	2008-2009
**Italy**	Deceleration in downward suicide rate trend: 2006 (2002, 2010)	2008-2009
**Japan**		2008-2009
**Poland**		None
**Republic of Korea**		None
**Spain**	Temporary upturn: 2011 (2008, 2012)	2008-2013

Fisher's exact test comparing experiencing a recession with rising suicide rates: p=1.00

**Table 3:** **Levels of, and changes in, Gini index for the eleven study countries** (hypothesis: if income inequality influences suicide rates, rises in suicide rates will have occurred in countries with greater absolute, or increasing, income inequality)

Country	Change in Gini index, 2004-2014^a^ (rank^c^)	Gini index, 2008^b^ (rank^d^)
*Countries where suicide rates are rising*		
Australia	+2.7 (2)	35.4 (2)
Canada	+0.3 (7)	33.8 (5=)
UK	-2.0 (9)	34.1 (4)
USA	+0.5 (5)	41.1 (1)

*Countries where suicide rates are not rising*		
France	+1.7 (3)	33.0 (7)
Germany	+0.7 (4)	31.2 (10)
Italy	+0.4 (6)	33.8 (5=)
Japan	-	32.1 (9)
Poland	-5.2 (10)	33.5 (6)
Republic of Korea	-0.1 (8)	32.3 (8)
Spain	+2.8 (1)	34.2 (3)
Wilcoxen rank sum test (comparing countries with and without rises)	z=-0.29 p=0.77	z=-2.45 p=0.01

a Due to restricted data availability, change in Gini calculated between the following dates for four countries: Canada, USA & Germany (2004-2013), Republic of Korea (2006-2012)

b Due to restricted data availability, 2007 Gini index provided for Canada & USA

c 1=greatest increase in Gini index; 10=greatest decrease in Gini index

d 1=highest Gini index; 10=lowest Gini index

Japan and France experienced a recession but did not show evidence of a concurrent rise in suicide rates. Japan and Republic of Korea's previously rising suicide rates reversed soon after the 2008 economic recession. Poland neither experienced a recession nor rises in suicide rates. Furthermore, based on their 95% confidence intervals, Spain and Germany's temporary upturn in suicide rates may have occurred around the time of the 2008 economic recession, as may the deceleration in Italy's downward suicide rate trend. Nonetheless, we found no statistical evidence that countries with rising suicide rates were more likely to have been affected by the 2008 global economic recession (Fisher’s exact test: p=0.49). This was unchanged when we reclassified Australia as not having experienced a recession (Fisher's exact test: p=1.00), and also when we excluded the two countries that experienced temporary upturns in suicide rates (Germany and Spain) (Fisher's exact test: p=1.00).

There is evidence that absolute GDP per capita in 2008 (the year the global recession began), is higher in countries with rising suicide rates (Wilcoxon rank sum test z=-2.27; p=0.02). Japan and the Republic of Korea experienced prolonged rises in suicide rates during the study period, followed by declines from 2009 onwards. Our findings were similar in a sensitivity analysis excluding these two countries (Wilcoxon rank sum test z=-1.96; p=0.05).

### 3.2 Suicide trends in relation to GINI index of income inequality

Three of the four countries with rising suicide rates experienced rises in income inequality between 2004-2014, but so too did at least four of the seven countries that do not have rising suicide rates (Wilcoxon rank sum test z=-0.29; p=0.77) (Table 3). There was however evidence that countries with rising suicide rates had higher levels of income inequality in 2008, at the midpoint of the time series (Wilcoxon rank sum test z=-2.45; p=0.01). This finding was unchanged when Japan and Republic of Korea were excluded from the analysis (z=-2.12; p=0.03).

### 3.3 Association of suicide trends with social media use

Rises in the proportion of young people using social media for ≥3 hours daily between 2013-2017 were seen in all of the countries studied except Italy (Table 4. Web Appendix 6). There was no statistical evidence that the change between 2013-2017 differed between countries with and without rising suicide rates (Wilcoxon rank sum test z=- 0.76; p= 0.45).

**Table 4:** **Use and** t**rends in use, of social media for the eleven study countries** (hypothesis: if rises in suicide rates are associated with growth in social media use, all countries will have experienced a rise in the incidence of suicide in 15-24 year olds, and increases in suicide will be greatest in countries that have experienced the greatest increases in social media use)

Country	Social Media trends, 2013-2017 (% using for ≥3hr/day)	% using social media for ≥3 hours a day, 2013 (rank⁎)
*Countries where suicide rates are rising*		
Australia	↑ (24.6 to 34.1%)	24.6 (5)
UK	↑ (26.4 to 39.8%)	26.4 (3)
USA	↑ (26.5 to 33.5%)	26.5 (2)
Canada	↑ (22.5 to 36.0%)	22.5 (6)
*Countries where suicide rates are not rising*		
France	↑ (20.7 to 26.3%)	20.7 (7)
Germany	↑ (18.8 to 23.4%)	18.8 (9)
Italy	↔ (32.0 to 32.6%)	32.0 (1)
Japan	↑ (2.2 to 17.3%)	2.2 (11)
Poland	↑ (20.5 to 28.8%)	20.5 (8)
Republic of Korea	↑ (7.2 to 20.9%)	7.2 (10)
Spain	↑ (25.7 to 34.3%)	25.7 (4)

Wilcoxon rank sum test (comparing countries where rates are and are not rising)	z= -0.76 (p=- 0.45)	z=-1.51 (p=-0.13)

⁎ 1= highest use; 11=lowest use

There was also no evidence that the proportion of 15-24 year olds using social media for ≥3 hours per day in 2013 was higher in the countries where suicide rates are rising (Wilcoxon rank sum test z=-1.51; p=0.13) (Table 4). The same pattern was observed when investigating the percentage using social media for ≥1 (z= -1.13; p=0.26) and ≥6 hours (z= -1.13; p=0.26) each day (Web appendix 7).

The two countries that have experienced the greatest declines in youth suicide in recent years, Japan and Republic of Korea, both had the lowest levels of social media use. When we excluded these two countries from our analysis, there was some evidence that the four countries experiencing rises in youth suicide have had the greatest increases in social media use between 2013-17 (Wilcoxon z=-1.96; p=0.05), but there was no evidence that they differed in relation to increases in use up to 2013 (z=-0.98; p=0.33).

## 4 Discussion

### 4.1 Key findings

Four of the eleven high-income countries studied are experiencing a rise in suicide rates amongst 15-24 year olds. These four countries were the only predominantly English-speaking countries in the study. The year these increases in suicide rates appeared to begin varied from country to country, ranging from 2003 to 2009. There is evidence that the rate of rise in suicide rates has recently accelerated in the UK and USA.

Levels of income inequality and GDP at the mid-point in the time-series, but not changes in income inequality, were higher in countries where youth suicide rates are rising than in countries with no such rise. There was inconsistent evidence of an association between the occurrence or timing of a recession and national trends in youth suicide. Although statistical testing did not find evidence for an association, this may have been due to the small number of countries included. In three of the four countries with rising suicide rates the 95% confidence intervals for the onset of the rise included the year of the onset of the recession (2008), and there was a short-lived upturn in suicide rates around 2008 in two other countries. Yet three of the countries that experienced a recession had no upturn in suicide rates.

There was no evidence that the proportion of 16-24 year olds using social media for three or more hours per day in 2013 was any higher in the four countries where suicide rates are rising than in the seven countries where they are not. There was also no evidence to indicate an association between population-level increases between 2013-2017 in time spent on social media and the rises in youth suicide, although there was some evidence of an association in our sensitivity analysis when we excluded the two countries with the greatest declines in suicide rates and lowest rates of social media use (Japan and Republic of Korea).

### 4.2 Findings in the context of the wider literature

Rises in youth suicide rates have previously been reported in England and Wales (Bould et al., 2019), the USA (Ruch et al., 2019) and Australia (Stefanac et al., 2019). The years studied, the geographic regions (UK vs. England and Wales), the precise ages studied, and the statistical approaches were different in this study compared to these earlier analyses. However, the timing of the increase reported in these studies, around 2010 in England and Wales, 2007 in the USA, and 2004 (females only) in Australia, fall within the 95% confidence intervals for years during which changes in trends were seen in our findings.

Studies in Australia, Canada, the UK and USA indicate that the rises in suicide in young people have occurred in parallel with other signals of deteriorating youth mental health, including rises in self-reported depression symptoms, suicide attempts and hospital admissions for self-harm (McManus et al., 2016; Mitchell et al., 2018; Twenge et al., 2019; Wiens et al., 2020).

The rise in youth suicide rates seen in Canada contrast with relatively stable trends in all-age suicide rates in Canada between 2004 and 2015 (Alicandro et al., 2019). Australia, the UK and USA have all experienced rises in overall suicide rates over this period, although the timing of changes in these trends in overall suicide differ to those in youth suicides in the UK and USA (Alicandro et al., 2019; Office for National Statistics, 2019).

Across 54 countries globally, suicide rates increased in many age groups, including those aged 15-24 years, following the 2008 global economic crisis (Chang et al., 2013). In keeping with the temporary upturns from around 2010 identified in two countries in our study (Spain and Germany), recession-related suicides began to decline in several countries in conjunction with indications of economic recovery (Alicandro et al., 2019).

We found that the absolute level of income inequality in 2008 was associated with rises in suicide rates, but the change in income inequality between 2004-2014 was not. This difference is likely due to the relative stability of income inequality over time. Spearman's rank-order correlation was positive between the Gini index values for 2004 compared with 2014 (rs=0.60, p=0.07). As a result, the 2008 measure is likely to provide a better indicator of relative income inequality, although it should be acknowledged that its specificity in relation to the timing of the suicide rate rises is limited. Rather than driving up suicide rates in itself, income inequality may provide a context in which other exposures may have greater impact. However, evidence regarding the association between income inequality and suicide rates is mixed, with multi-country analyses revealing a possible association between lower levels of income inequality and increased suicide rates (Lynch et al., 2001). Nonetheless, there is evidence indicating an association between higher income inequality and negative mental health outcomes (Ribeiro et al., 2017). Similarly, we found evidence that the countries with higher GDPs in 2008 were those that are experiencing rises in suicide. It is possible that high levels of income inequality in rich countries contribute to the suicide rate rises.

There has been intense speculation in the scientific literature and popular media, about the influence of social media on young people's mental health. However, the current evidence base is relatively weak. A recent systematic review of adolescent mental health outcomes in relation to social media use identified almost exclusively cross-sectional studies, and findings were mixed in relation to time spent on social media (Keles et al., 2020). Prospective studies, not limited to adolescents, have also obtained varied results regarding an association between social media use and wellbeing measures (Hunt et al., 2018; Orben and Przybylski, 2019; Shakya and Christakis, 2017; Tromholt, 2016). Our time trends study does not find any strong evidence of an association between time spent on social media and rises in youth suicide rates at a population level.

### 4.3 Strengths and limitations

Our study is the first to comprehensively describe trends of youth suicide reported in the most populous high-income countries around the world. Previous analyses have mainly focused on single countries. This may lead to publication bias as researchers will tend to focus on countries that have experienced adverse trends or marked changes in rates. Our study selected countries a priori based on their population size, and only one eligible country (Saudi Arabia) did not have age-specific suicide data available. Preceding cross-national analyses of youth suicide rates have not investigated trends (Glenn et al., 2019), have completed follow up before or during the period in which many countries experienced upturns in suicide rates (Innamorati et al., 2010; Kõlves and De Leo, 2016), and/or included low- or middle-income countries where drivers are likely to be different (Glenn et al., 2019; Innamorati et al., 2010; Kõlves and De Leo, 2016; McLoughlin et al., 2015).

There are several limitations to consider when interpreting our findings. First, due to constraints in the availability of data, we were only able to investigate three a priori factors potentially linked to recent rises in youth suicide, and were not able to adjust for their effects on each other. For example, we only had concurrent data for social media and GDP for five years of the 17-year series. Furthermore, Gini index data were incomplete, making multivariable time series analysis impossible. Other factors which may be associated with rises in youth suicide (e.g. concern about the planet) are more challenging to quantify with routinely available data. To the best of our knowledge no data source provides reliable and comparable age-specific data on factors such as divorce and alcohol or drug misuse. Yet these other factors may explain the associations we observed or independently have influenced the trends in suicide rates.

Second, our dichotomization of countries based on whether or not they have rising suicide rates may also have introduced a bias, which might mask subtle differences, particularly in relation to economic exposures. For example, Japan and the Republic of Korea experienced rises in suicide rates during the early years of the study period, followed by steep declines. Nonetheless, our findings were unchanged when Japan and the Republic of Korea were excluded from the analyses.

Third, we considered the influence of an economic recession by identifying a fall in GDP. This approach does not take into account differences in national responses to the recession, such as the implementation of austerity measures (Reeves et al., 2015).

Fourth, self-reported time spent on social media only provides a crude indicator of its use. Evidence suggests that passive consumption of social media content may be negatively associated with mental health, possibly via the mechanisms of upward social comparisons and feelings of envy (Verduyn et al., 2015). In contrast, active social networking, including direct communication, has been associated with increased social capital and reduced loneliness (Verduyn et al., 2017). It may be that the type of social media content with which young people engage is more important for suicide-related outcomes than overall time spent using social media (George, 2019). Nonetheless time spent on social media generally equates to time not spent on alternative activities known to have beneficial impacts on mental health such as physical exercise and sleep (Viner et al., 2019).

Fifth, social media data for the study countries were only available for complete years from 2013 onwards, and the survey data are likely to provide at best a crude indication of time spent on social media. For example, as survey participants were recruited online, they may not be representative of the general population in countries with low or variable Internet penetration rates. Amongst the countries studied GlobalWebIndex estimates 2019 Internet penetration for the total (all age) population ranged from 62% (Italy) to 96% (UK), although these figures are likely to be higher for 16-24 year olds (GlobalWebIndex, 2019). It is reassuring that GlobalWebIndex's estimate of social media usage for 15-24 year olds in the UK (30.5% used it for three or more hours per day in 2015) is broadly consistent with the estimate for 14 year olds in the UK in 2014-16 from the Millennium cohort: 43% for girls and 22% for boys reported social media use for at least 3 hours daily (Kelly et al., 2018).

Finally, ecological studies such as this one cannot determine whether those who took their lives were themselves affected by the risk factors investigated. Nevertheless, ecological studies are an appropriate approach for studying the impact of population level exposures such as income inequality and economic recession.

## 5 Conclusions

Our analyses indicate that recent rises in youth suicide have occurred in all four of the most populous high-income predominantly English-speaking nations (Australia, Canada, UK and USA), which are also characterised as having higher levels of income inequality and GDP than the other countries studied. There was also a suggestion of a short-term impact of the global economic crisis on youth suicide. Due to a lack of comparable data, it was not possible to investigate the effect of other potentially important contributors to trends in population suicide rates, such as alcohol or drug misuse and parental marriage breakdown, including their relationship with the explanatory factors studied. These findings, therefore, provide preliminary evidence regarding possible contributory factors to guide further research, rather than a clear target to incorporate within suicide prevention strategies.

## Funding

D.G. and P.M. are supported by the NIHR Biomedical Research Centre at University Hospitals Bristol NHS Foundation Trust and the University of Bristol, England. The views expressed in this publication are those of the author(s) and not necessarily those of the NHS, the National Institute for Health Research or the Department of Health and Social Care. P.P.’s PhD Clinical Fellowship is funded by the MRC Addiction Research Clinical Training programme (MR/N00616X/1).

## CRediT authorship contribution statement

**Prianka Padmanathan:** Data curation, Formal analysis, Project administration, Writing - review & editing. **Helen Bould:** Supervision, Writing - review & editing. **Lizzy Winstone:** Writing - review & editing. **Paul Moran:** Writing - review & editing. **David Gunnell:** Conceptualization, Methodology, Formal analysis, Writing - original draft, Supervision, Writing - review & editing.

## Declaration of Competing Interests

Nil to declare

## Appendix Supplementary materials

Image, application 1

Image, application 2

Image, application 3

Image, application 4

Image, application 5

Image, application 6

Image, application 7
