# Supplementary material for: Social media use, economic recession and income inequality in relation to trends in youth suicide in high-income countries: a time trends analysis
Source: J Affect Disord. 2020 Oct 1;275:58–65. doi: 10.1016/j.jad.2020.05.057 (PMC7397515; doi:10.1016/j.jad.2020.05.057)
Supplement: Supplementary file 1 [file mmc1.docx]

**Web appendix 1: High-income countries by population**

*Yellow represents countries with populations under 20 million. Red represents exclusion from study due to lack of data.*

| High-income countries (World Bank, 2020: https://datahelpdesk.worldbank.org/knowledgebase/articles/906519-world-bank-country-and-lending-groups) | Total populations (World Bank, 2018: https://data.worldbank.org/indicator/sp.pop.totl) |
| --- | --- |
| United States | 327167434 |
| Japan | 126529100 |
| Germany | 82927922 |
| France | 66987244 |
| United Kingdom | 66488991 |
| Italy | 60431283 |
| Korea, Rep. | 51635256 |
| Spain | 46723749 |
| Poland | 37978548 |
| Canada | 37058856 |
| Saudi Arabia | 33699947 |
| Australia | 24992369 |
| Chile | 18729160 |
| Netherlands | 17231017 |
| Belgium | 11422068 |
| Greece | 10727668 |
| Czech Republic | 10625695 |
| Portugal | 10281762 |
| Sweden | 10183175 |
| Hungary | 9768785 |
| United Arab Emirates | 9630959 |
| Israel | 8883800 |
| Austria | 8847037 |
| Switzerland | 8516543 |
| Hong Kong SAR, China | 7451000 |
| Denmark | 5797446 |
| Singapore | 5638676 |
| Finland | 5518050 |
| Slovak Republic | 5447011 |
| Norway | 5314336 |
| New Zealand | 4885500 |
| Ireland | 4853506 |
| Oman | 4829483 |
| Panama | 4176873 |
| Kuwait | 4137309 |
| Croatia | 4089400 |
| Uruguay | 3449299 |
| Puerto Rico | 3195153 |
| Lithuania | 2789533 |
| Qatar | 2781677 |
| Slovenia | 2067372 |
| Latvia | 1926542 |
| Bahrain | 1569439 |
| Trinidad and Tobago | 1389858 |
| Estonia | 1320884 |
| Cyprus | 1189265 |
| Macao SAR, China | 631636 |
| Luxembourg | 607728 |
| Malta | 483530 |
| Brunei Darussalam | 428962 |
| Bahamas, The | 385640 |
| Iceland | 353574 |
| Barbados | 286641 |
| New Caledonia | 284060 |
| French Polynesia | 277679 |
| Channel Islands | 170499 |
| Guam | 165768 |
| Curacao | 159849 |
| Virgin Islands (U.S.) | 106977 |
| Aruba | 105845 |
| Seychelles | 96762 |
| Antigua and Barbuda | 96286 |
| Isle of Man | 84077 |
| Andorra | 77006 |
| Cayman Islands | 64174 |
| Bermuda | 63968 |
| Northern Mariana Islands | 56882 |
| Greenland | 56025 |
| St. Kitts and Nevis | 52441 |
| Faroe Islands | 48497 |
| Sint Maarten (Dutch part) | 40654 |
| Monaco | 38682 |
| Liechtenstein | 37910 |
| Turks and Caicos Islands | 37665 |
| St. Martin (French part) | 37264 |
| San Marino | 33785 |
| Gibraltar | 33718 |
| British Virgin Islands | 29802 |
| Palau | 17907 |
| Taiwan, China | Unavailable |
| **Total population** | **1186737873** |
| **Population of included countries** | **928920752** |
| **% of high-income countries covered** | **78.27514173** |
