# Supplementary material for: Social media use, economic recession and income inequality in relation to trends in youth suicide in high-income countries: a time trends analysis
Source: J Affect Disord. 2020 Oct 1;275:58–65. doi: 10.1016/j.jad.2020.05.057 (PMC7397515; doi:10.1016/j.jad.2020.05.057)
Supplement: Supplementary file 2 [file mmc2.docx]

**Web Appendix 2: Years during study period (2000-2017) for which data were available**

| **Country** | **Population**  World Bank, 2019 | **Suicide (15-24 yrs)**  WHO, Dec 2019 | **Social media use**  GlobalWebIndex | **GDP**  World Bank, 2017 | **Gini index**  World Bank, 2017 |
| --- | --- | --- | --- | --- | --- |
| Australia | 2000-2017 | 2000-2004  2006-2017 | 2013-2017 | 2000-2017 | 2001  2003  2004  2008  2010  2014 |
| Canada | 2000-2017 | 2000-2017 | 2013-2017 | 2000-2017 | 2000  2004  2007  2010  2013 |
| France | 2000-2017 | 2000-2016 | 2013-2017 | 2000-2017 | 2003-2015 |
| Germany | 2000-2017 | 2000-2017 | 2013-2017 | 2000-2017 | 2000-2011  2013  2015 |
| Italy | 2000-2017 | 2003-2016 | 2013-2017 | 2000-2017 | 2003-2015 |
| Japan | 2000-2017 | 2000-2017 | 2013-2017 | 2000-2017 | 2008 |
| Korea, Rep. | 2000-2017 | 2000-2017 | 2013-2017 | 2000-2017 | 2006  2008  2010  2012 |
| Poland | 2000-2017 | 2000-2017 | 2013-2017 | 2000-2017 | 2004-2015 |
| Spain | 2000-2017 | 2000-2017 | 2013-2017 | 2000-2017 | 2003-2015 |
| United Kingdom | 2000-2017 | 2001-2016 | 2013-2017 | 2000-2017 | 2004-2015 |
| United States | 2000-2017 | 2000-2017 | 2013-2017 | 2000-2017 | 2000  2004  2007  2010  2013  2016 |
