# Supplementary material for: Social media use, economic recession and income inequality in relation to trends in youth suicide in high-income countries: a time trends analysis
Source: J Affect Disord. 2020 Oct 1;275:58–65. doi: 10.1016/j.jad.2020.05.057 (PMC7397515; doi:10.1016/j.jad.2020.05.057)
Supplement: Supplementary file 3 [file mmc3.docx]

**Web Appendix 3: Sex-specific annual percent change (APC) in suicide rates and joinpoints (JP) for modelled trends in suicide rates in 15-24 year olds in high-income countries**

| **Country / sex** | **Segment 1**  **APC (95% CI) suicide rate** | **Join point 1**  **Year (95% CI)** | **Segment 2**  **APC (95% CI) suicide rate** | **Join point 2**  **Year (95% CI)** | **Segment 3**  **APC (95% CI) suicide rate** |  |  |
| --- | --- | --- | --- | --- | --- | --- | --- |
| **Australia F** | -16.4 (-41.4, 19.3) | 2002 (2002, 2008) | 3.6 (2.1, 5.2) |  |  |  |  |
| **Australia M** | -4.8 (-6.5, -3.2) | 2009 (2007, 2011) | 4.5 (2.4, 6.6) |  |  |  |  |
|  |  |  |  |  |  |  |  |
| **Canada F** | 1.7 (0.7, 2.7) |  |  |  |  |  |  |
| **Canada M** | -1.5 (-2.3, -0.7) |  |  |  |  |  |  |
|  |  |  |  |  |  |  |  |
| **France F** | -2.2 (-3.2, -1.1) |  |  |  |  |  |  |
| **France M** | -3.3 (-4.1, -2.5) |  |  |  |  |  |  |
|  |  |  |  |  |  |  |  |
| **Germany F** | -1.1 (-2.1, -0.0) |  |  |  |  |  |  |
| **Germany M** | -1.3 (-7.5, 5.4) | 2002 (2002, 2004) | -7.2 (-9.2, -5.1) | 2007 (2005, 2008) | 0.8 (-3.1, 4.8) | 2011 (2009, 2014) | -3.6 (-5.0, -2.2) |
|  |  |  |  |  |  |  |  |
| **Italy F** | -1.8 (-3.2, -0.3) |  |  |  |  |  |  |
| **Italy M** | -2.9 (-3.9, -1.9) |  |  |  |  |  |  |
|  |  |  |  |  |  |  |  |
| **Japan F** | 5.5 (2.7, 8.3) | 2008 (2006, 2010) | -4.6 (-6.9, -2.2) |  |  |  |  |
| **Japan M** | 3.8 (2.7, 4.8) | 2010 (2008, 2013) | -2.8 (-4.5, -0.9) |  |  |  |  |
|  |  |  |  |  |  |  |  |
| **Poland F** | -0.0 (-1.2, 1.1) |  |  |  |  |  |  |
| **Poland M** | 0.5 (0.0, 0.9) | 2014 (2013, 2015) | -7.8 (-13.0, -2.4) |  |  |  |  |
|  |  |  |  |  |  |  |  |
| **Republic of Korea F** | 12.2 (7.0, 17.7) | 2008 (2006, 2010) | -7.4 (-10.9, -3.8) |  |  |  |  |
| **Republic of Korea M** | 5.5 (2.0, 9.1) | 2009 (2006, 2013) | -1.7 (-5.2, 2.0) |  |  |  |  |
|  |  |  |  |  |  |  |  |
| **Spain F** | 14.1 (-3.0, 34.3) | 2003 (2002, 2006) | -8.8 (-13.1, -4.4) | 2011 (2008, 2012) | 35.9 (-3.9, 92.2) | 2014 (2012, 2015) | -14.8 (-28.5, 1.5) |
| **Spain M** | -2.7 (-3.9, -1.4) |  |  |  |  |  |  |
|  |  |  |  |  |  |  |  |
| **UK F** | -2.0 (-4.4, 0.5) | 2013 (2005, 2014) | 20.5 (-2.3, 48.4) |  |  |  |  |
| **UK M** | -14.1 (-19.6, -8.2) | 2003 (2002, 2006) | 1.3 (0.5, 2.0) |  |  |  |  |
|  |  |  |  |  |  |  |  |
| **USA F** | 1.9 (0.1, 3.8) | 2008 (2005, 2013) | 5.5 (4.3, 6.8) |  |  |  |  |
| **USA M** | -1.2 (-1.9, -0.4) | 2007 (2005, 2010) | 1.6 (0.7, 2.6) | 2014 (2012, 2015) | 7.6 (4.9, 10.3) |  |  |
