# Supplementary figures and images for: Social media use, economic recession and income inequality in relation to trends in youth suicide in high-income countries: a time trends analysis
Source: J Affect Disord. 2020 Oct 1;275:58–65. doi: 10.1016/j.jad.2020.05.057 (PMC7397515; doi:10.1016/j.jad.2020.05.057)

**
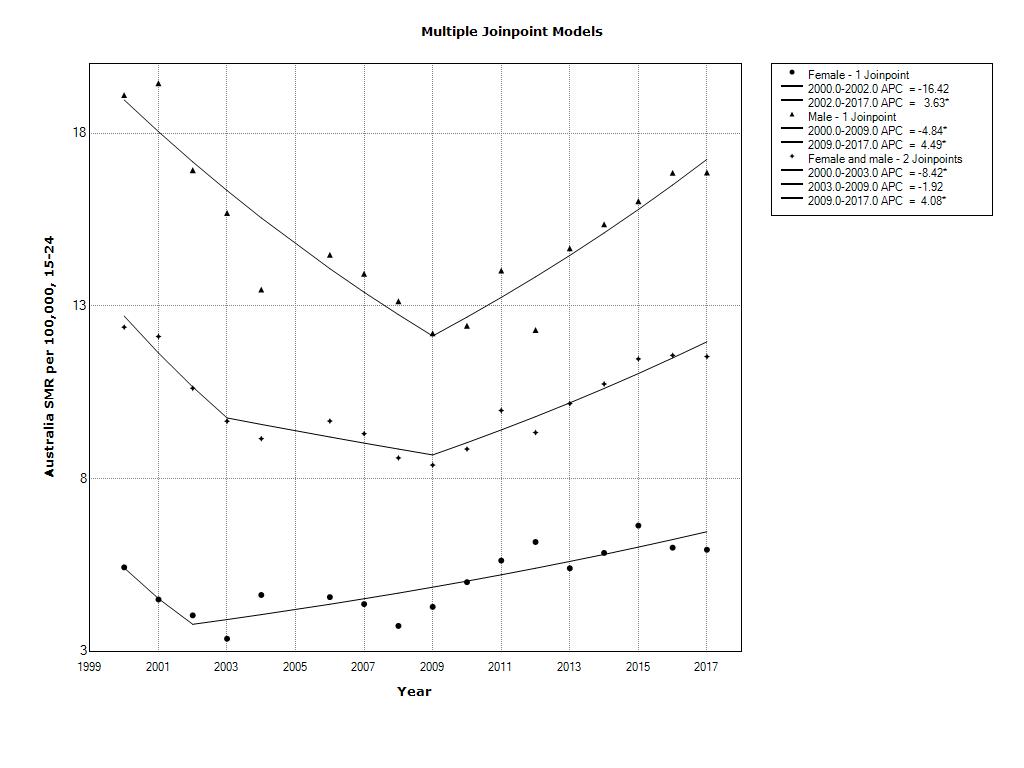
Web appendix 4: Overall and sex-specific suicide rates in 15-24 year olds in high-income countries
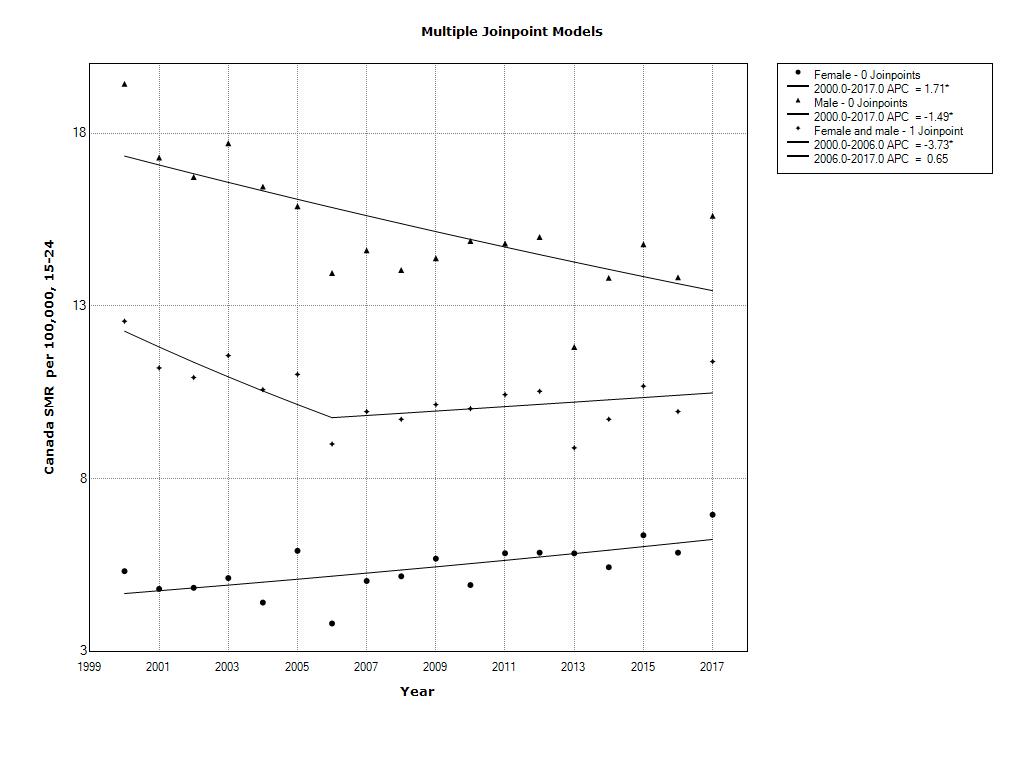

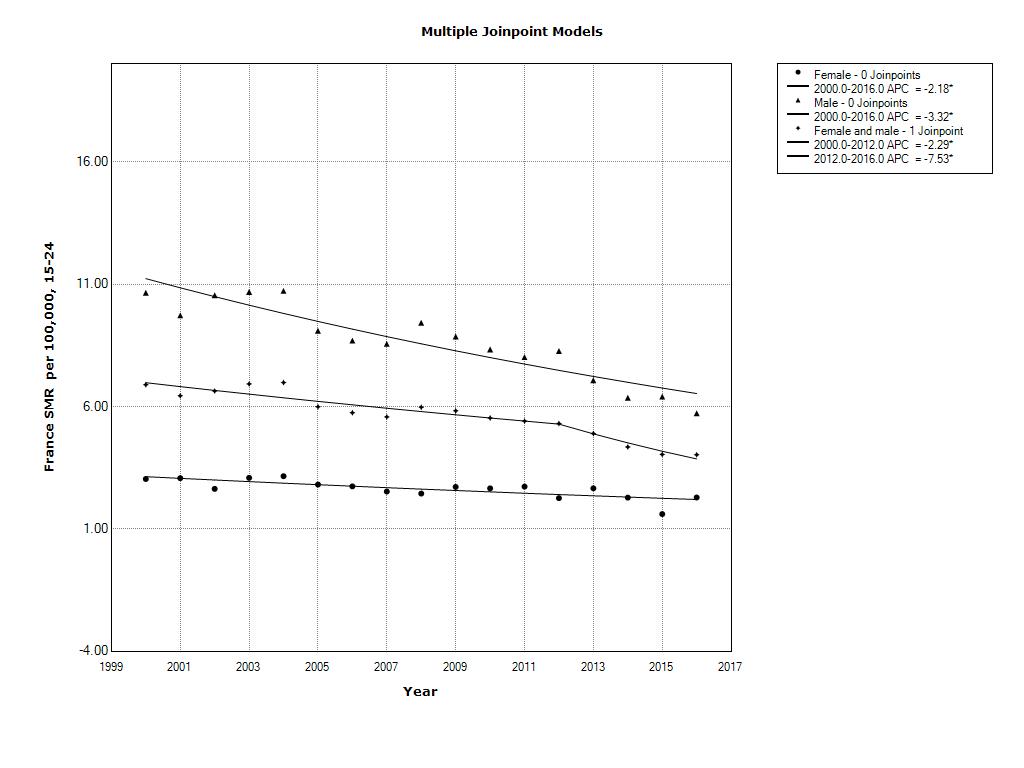

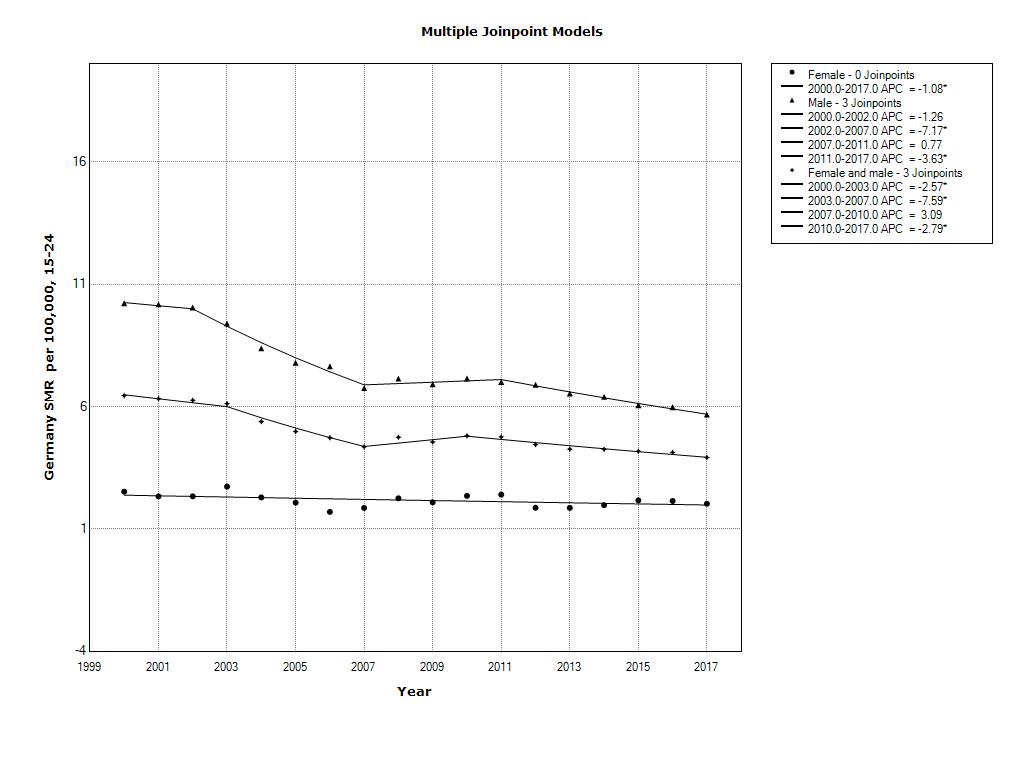

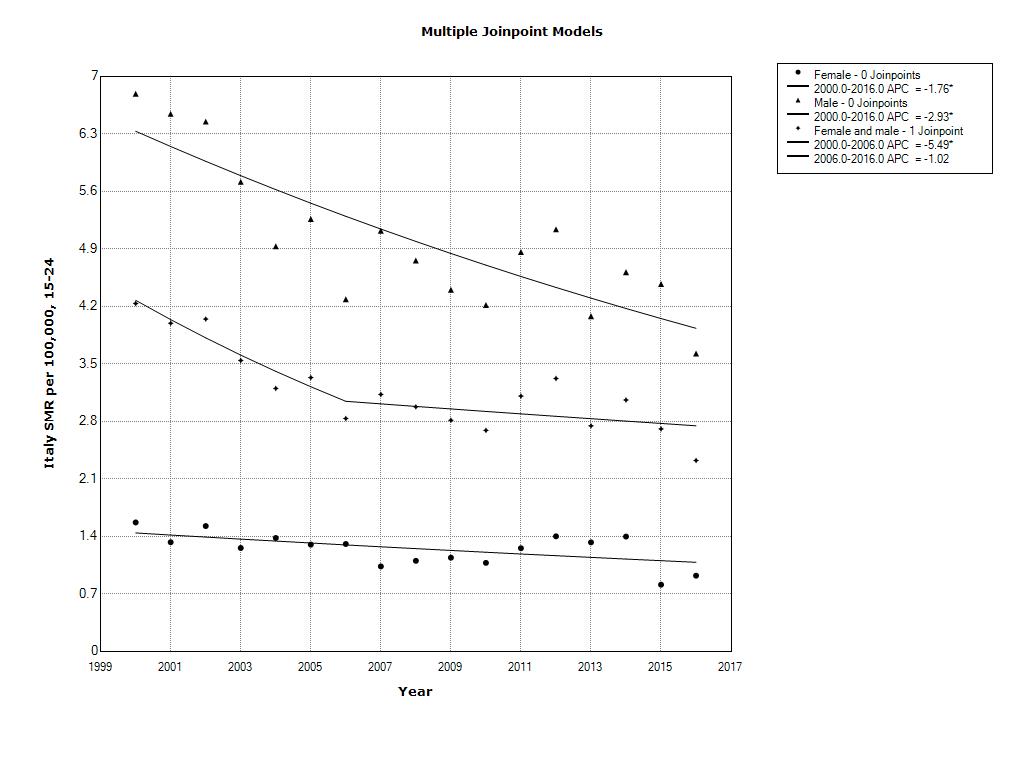

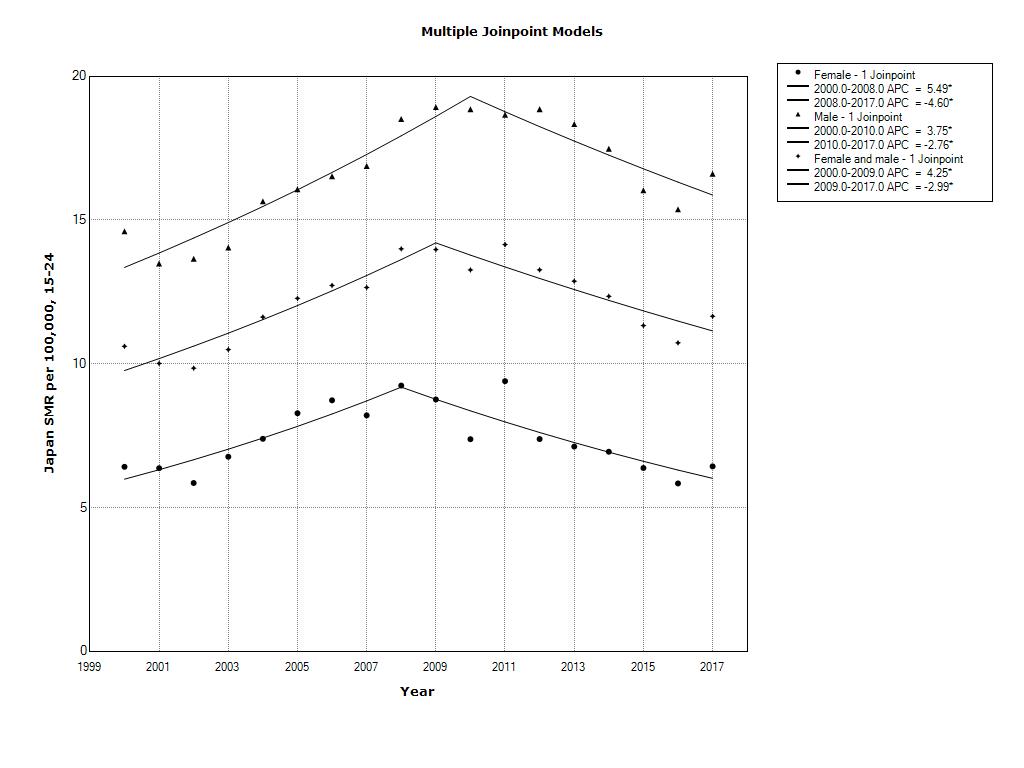

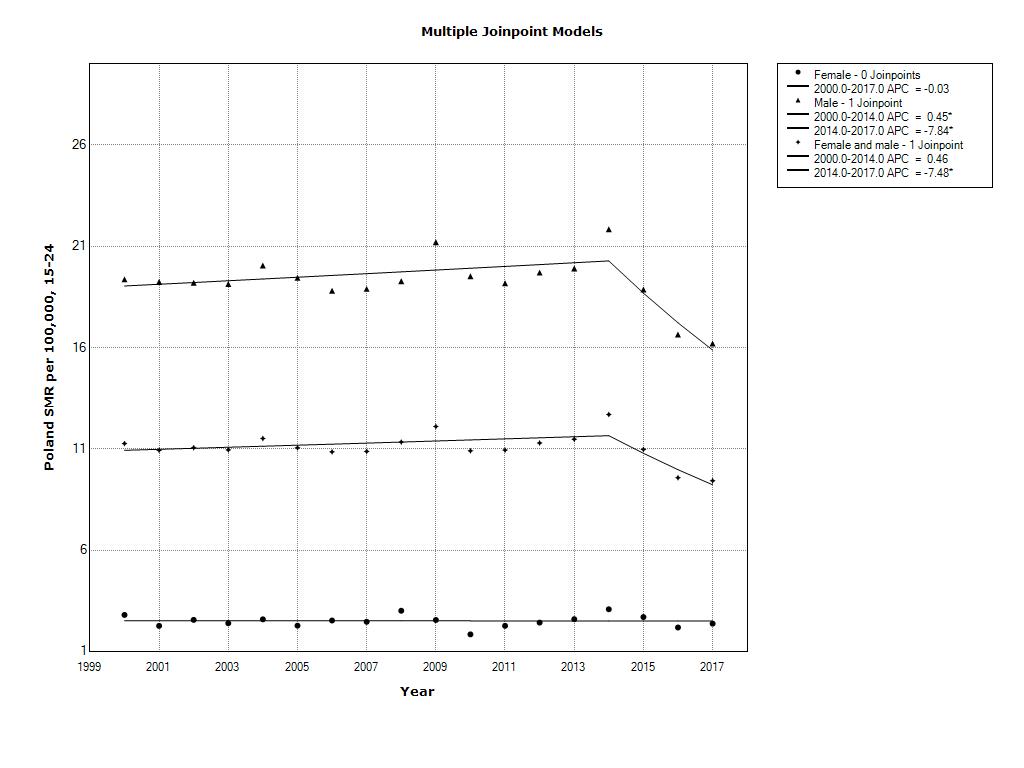

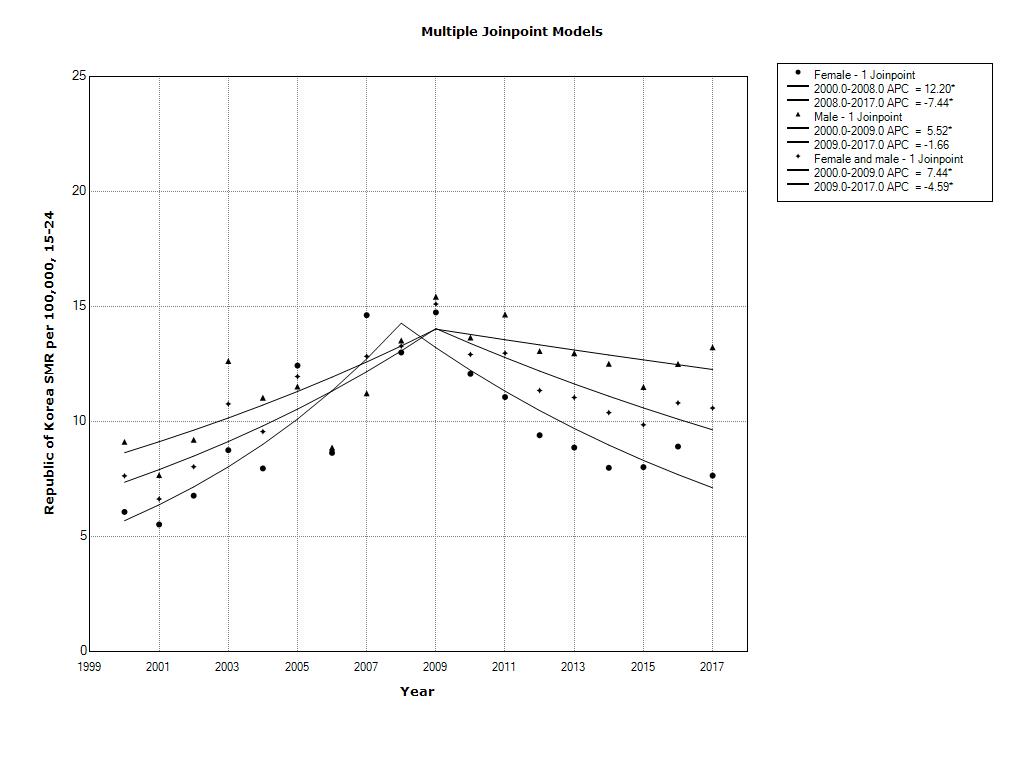

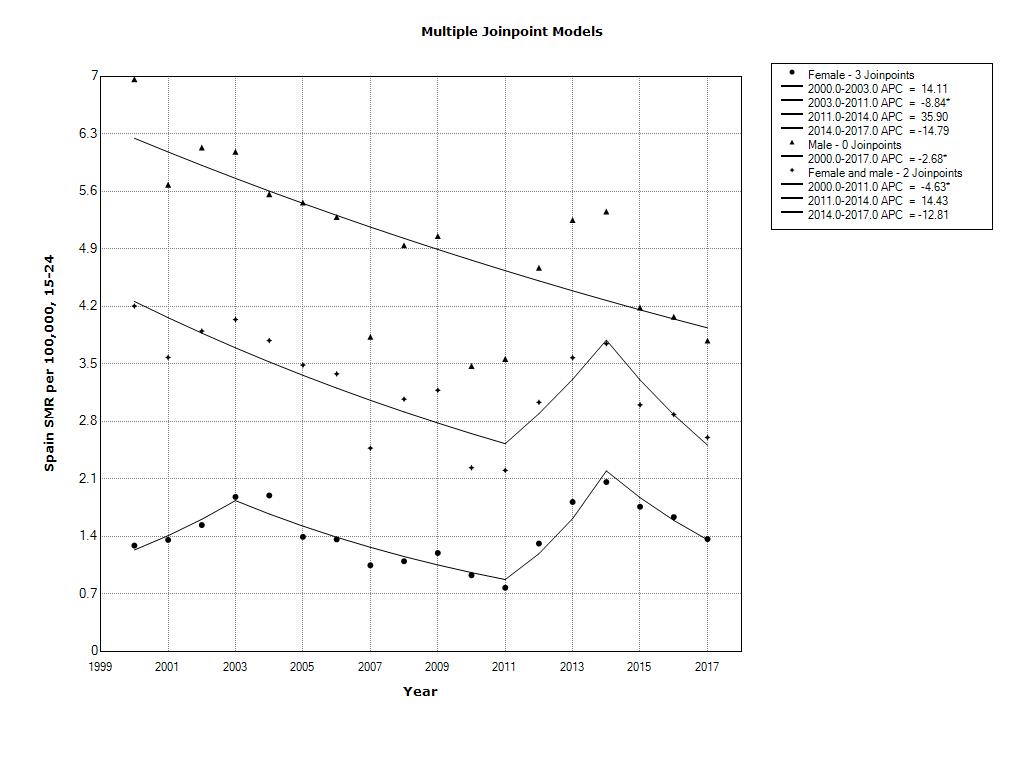

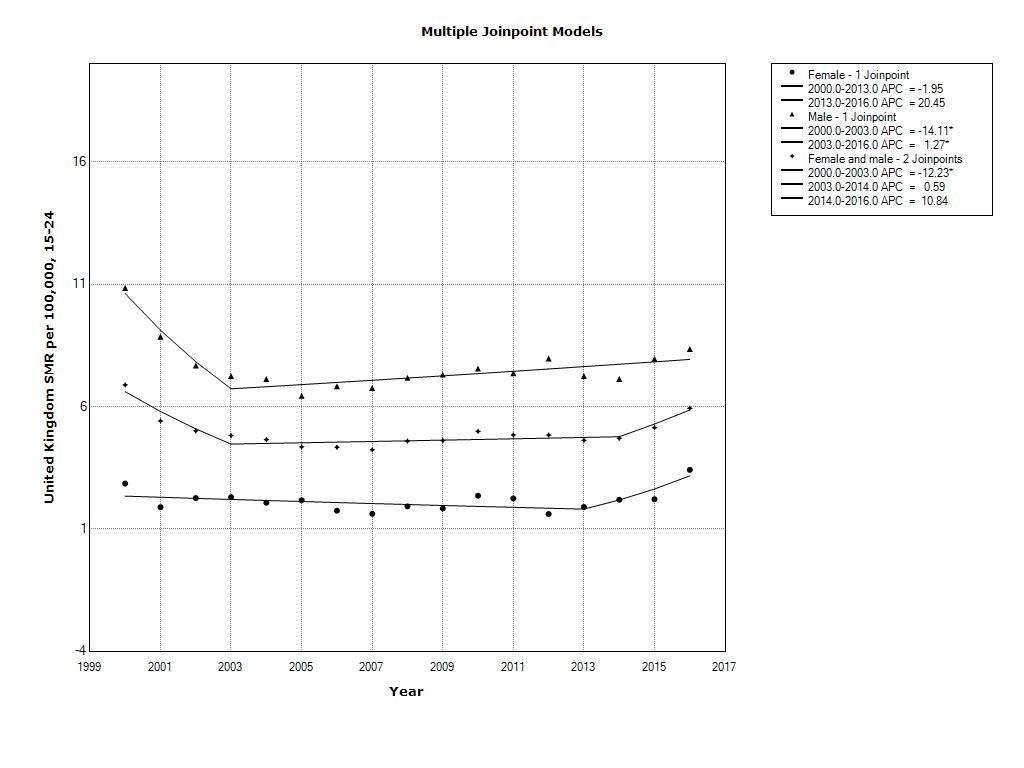

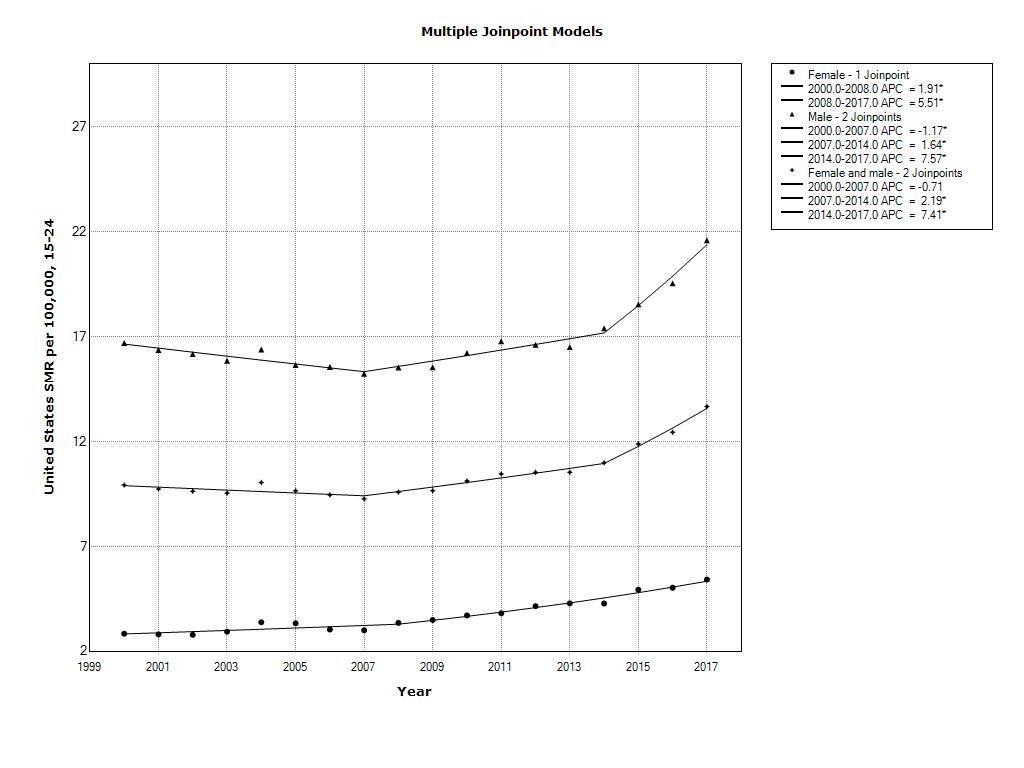
**

Supplement: Supplementary file 4 [file mmc4.docx]
