# Supplementary material for: Social media use, economic recession and income inequality in relation to trends in youth suicide in high-income countries: a time trends analysis
Source: J Affect Disord. 2020 Oct 1;275:58–65. doi: 10.1016/j.jad.2020.05.057 (PMC7397515; doi:10.1016/j.jad.2020.05.057)
Supplement: Supplementary file 5 [file mmc5.docx]

**Web appendix 5: GDP per capita**

Trends in GDP per capita in high-income countries where suicide rates are rising

Trends in GDP per capita in high-income countries where suicide rates are not rising
