# Supplementary material for: Social media use, economic recession and income inequality in relation to trends in youth suicide in high-income countries: a time trends analysis
Source: J Affect Disord. 2020 Oct 1;275:58–65. doi: 10.1016/j.jad.2020.05.057 (PMC7397515; doi:10.1016/j.jad.2020.05.057)
Supplement: Supplementary file 6 [file mmc6.docx]

**Web appendix 6: Time spent using social media (≥3 hours a day)**

Proportion of 16-24 year olds using social media for ≥3 hours a day in high-income countries where suicide rates are rising

|  |  |
| --- | --- |
|  |  |
|  |  |

Proportion of 16-24 year olds using social media for ≥3 hours a day in high-income countries where suicide rates are not rising

|  |  |
| --- | --- |
|  |  |
|  |  |
