# Supplementary material for: Social media use, economic recession and income inequality in relation to trends in youth suicide in high-income countries: a time trends analysis
Source: J Affect Disord. 2020 Oct 1;275:58–65. doi: 10.1016/j.jad.2020.05.057 (PMC7397515; doi:10.1016/j.jad.2020.05.057)
Supplement: Supplementary file 7 [file mmc7.docx]

**Web appendix 7: Time spent using social media (≥1 hour or ≥6 hours a day)**

Proportion of 16-24 year olds using social media for ≥1 hour or ≥6 hours a day in high-income countries where suicide rates are rising

Proportion of 16-24 year olds using social media for ≥1 hour or ≥6 hours a day in high-income countries where suicide rates are not rising
